# Influenza and respiratory syncytial virus in infants study (IRIS) of hospitalized and non-ill infants aged <1 year in four countries: study design and methods

**DOI:** 10.1186/s12879-017-2299-7

**Published:** 2017-03-22

**Authors:** Mark G. Thompson, Danielle R. Hunt, Ali K. Arbaji, Artan Simaku, Veronica L. Tallo, Holly M. Biggs, Carolyn Kulb, Aubree Gordon, Ilham Abu Khader, Silvia Bino, Marilla G. Lucero, Eduardo Azziz-Baumgartner, Pat Shifflett, Felix Sanchez, Basima I. Marar, Ilirjana Bakalli, Eric A. F. Simões, Min Z. Levine, Jennifer K. Meece, Angel Balmaseda, Tareq M. Al-Sanouri, Majlinda Dhimolea, Joanne N. de Jesus, Natalie J. Thornburg, Susan I. Gerber, Lionel Gresh, on behalf of IRIS Network, Brett Whitaker, Azaibi Tamin, Teresa C. T. Peret, Meredith McMorrow, Rebecca Fink, Laura Edwards, Guillermina Kuan, Nery Sanchez, Sergio Ojeda, Karla Patricia Membreño, Aktham Haddadin, Mahmoud Al-Gazo, Diozele Sanvictores, Edelwisa S Mercado, Maribeth Jimenez, Ma Azucena Redillas, Analyn Suaffield, Marilie Dagupan, Jenilyn Lungat, Amalia Penaranda, Lei Lanna Mendoza-Dancel, Karen Lana Cruz

**Affiliations:** 1 Influenza Division, Centers for Disease Control and Prevention (CDC), Atlanta, GA USA; 2 Abt Associates, Inc, Atlanta, GA USA; 3 The Eastern Mediterranean Public Health Network (EMPHNET), Amman, Jordan; 4 Department of Epidemiology & Control of Infectious Diseases, Institute of Public Health, Tirana, Albania; 5 Research Institute for Tropical Medicine, Department of Health, Muntinlupa City, Metro Manila Philippines; 6 Division of Viral Diseases, CDC, Atlanta, GA USA; 7 Department of Epidemiology, School of Public Health, University of Michigan, Ann Arbor, MI USA; 8 Hospital Infantil Manuel de Jesús Rivera, Ministry of Health, Managua, Nicaragua; 9 Al-Bashir Hospital, Amman, Jordan; 10 Pediatric Hospital, University Hospital Centre, Tirana, Albania; 11 Section of Infectious Diseases, Department of Pediatrics, University of Colorado School of Medicine, Aurora, Colorado USA; 12 Center for Global Health, Department of Epidemiology, Colorado School of Public Health, Aurora, Colorado USA; 13 Marshfield Clinic Research Foundation, Marshfield, WI USA; 14 Laboratorio Nacional de Virología, Centro Nacional de Diagnóstico y Referencia, Ministry of Health, Managua, Nicaragua; 15 Department of Epidemiology & Control of Infectious Diseases, Virology Laboratory, Institute of Public Health, Tirana, Albania; 16 Sustainable Sciences Institute, Managua, Nicaragua; 17 Influenza Division, MS A-32, National Center for Immunization and Respiratory Disease, Centers for Disease Control and Prevention, 1600 Clifton Road, NE, Atlanta, GA 30333 USA

**Keywords:** Infant, Influenza, Respiratory syncytial virus, Hospital, Burden, Serology

## Abstract

**Background:**

This multi-country prospective study of infants aged <1 year aims to assess the frequency of influenza virus and respiratory syncytial virus (RSV) infections associated with hospitalizations, to describe clinical features and antibody response to infection, and to examine predictors of very severe disease requiring intensive care.

**Methods/Design:**

We are enrolling a hospital-based cohort and a sample of non-ill infants in four countries (Albania, Jordan, Nicaragua, and the Philippines) using a common protocol. We are currently starting year 2 of a 2- to 3-year study and will enroll approximately 3,000 infants hospitalized for any acute illness (respiratory or non-respiratory) during periods of local influenza and/or RSV circulation. After informed consent and within 24 h of admission, we collect blood and respiratory specimens and conduct an interview to assess socio-demographic characteristics, medical history, and symptoms of acute illness (onset ≤10 days). Vital signs, interventions, and medications are documented daily through medical record abstraction. A follow-up health assessment and collection of convalescent blood occurs 3-5 weeks after enrollment. Influenza and RSV infection is confirmed by singleplex real time reverse transcriptase polymerase chain reaction (rRT-PCR) assays. Serologic conversion will be assessed comparing acute and convalescent sera using hemagglutination inhibition assay for influenza antibodies and enzyme-linked immunosorbent assay (ELISA) for RSV. Concurrent with hospital-based enrollment, respiratory specimens are also being collected (and tested by rRT-PCR) from approximately 1,400 non-ill infants aged <1 year during routine medical or preventive care.

**Discussion:**

The *Influenza and RSV in Infants Study* (IRIS) promises to expand our knowledge of the frequency, clinical features, and antibody profiles of serious influenza and RSV disease among infants aged <1 year, quantify the proportion of infections that may be missed by traditional surveillance, and inform decisions about the potential value of existing and new vaccines and other prevention and treatment strategies.

## Background

International rates of hospitalization due to acute upper and lower respiratory disease are highest among infants aged <1 year old [1–3]. Influenza virus [1, 2, 4–6] and respiratory syncytial virus (RSV) [7, 8] infections are recognized as leading contributors to this burden; yet, existing studies typically underestimate the frequency of severe influenza and RSV diseases that require hospitalization among infants for multiple reasons. First, clinical complications from influenza virus infections, such as pneumonia and bronchiolitis, often occur days after the primary infection when molecular diagnostics may be less sensitive to detect reduced virus shedding by the time of hospitalization.^1^ However, as demonstrated by a study in Thailand, serologic diagnostics may be able to detect antibody seroconversion following influenza infection even among hospitalized pneumonia patients who test negative for influenza using molecular diagnostics [9–11]. Likewise, serology may be a complementary tool to molecular diagnostics in determining recent RSV infections [12, 13].

Second, existing surveillance platforms often overlook non-febrile infections and non-respiratory manifestations of influenza and RSV infection [14–17]. For example, in a study of children aged <3 years presenting with fever to emergency departments in France, about 1 in 3 children with laboratory-confirmed influenza presented with a non-respiratory disease (including febrile seizures, sepsis-like syndrome, and gastroenteritis) [14]. Likewise, RSV is typically considered an etiologic agent in respiratory tract infections during the RSV season [18, 19], but it is also an important cause of apnea in preterm infants [20–22] and can present with shock and as a sepsis-like syndrome in young infants [23–25]. Additionally, both influenza and RSV infections often presents without fever in infants [7, 17, 26]. Thus, the true burden and clinical manifestations of both influenza and RSV among infants might be under-represented if subjects are selected for pathogen testing among those with only respiratory symptoms or only fever. Finally, confidence in the etiologic role of influenza and RSV in infant hospitalization requires comparative information on the prevalence of these infections among non-ill infants at the same time and from the same communities, which is often overlooked in epidemiologic studies of this population [27].

This U.S. Centers for Disease Control and Prevention (US CDC) funded multi-site prospective study will enroll approximately 3,000 infants aged <1 year hospitalized for any acute illness and a comparison sample of approximately 1,400 non-ill infants.^2^ Here we provide an overview of the design and methods of the *Influenza and RSV in Infants Study* (IRIS) currently starting year 2 of a 2- to 3-year study in Albania, Jordan, Nicaragua, and the Philippines. Our study aims are to assess the frequency of influenza- and RSV-associated hospitalizations (i.e., severe disease) for respiratory and non-respiratory diseases, describe the clinical features of these infections and the predictors of intensive care (i.e., very severe disease), and describe the antibody response to influenza virus and RSV infections. Table 1 lists the knowledge gaps we identified within each of these aims and the study features intended to address these gaps.

**Table 1:** Study goals and features intended to address specific knowledge gaps

A. Assess the frequency of influenza- and RSV-associated hospitalizations among infants ages <1 year old	
Knowledge Gap	Study Feature
Few studies have examined influenza and RSV hospitalizations outside of high-income countries.	Enroll patients in study sites located in four diverse middle-income countries (Albania, Jordan, Nicaragua, and The Philippines).
Studies often enroll only during peak periods of virus circulation.	Enroll patients during an extended period to take into account prolonged and overlapping periods of influenza and RSV circulation.
Typical severe acute respiratory illness (SARI) surveillance strategies use highly specific case definitions that overlook non-respiratory and non-febrile manifestations of disease.	Enroll admissions due to any acute (respiratory and non-respiratory) illnesses; describe the clinical diagnoses associated with influenza and RSV infections.
Secondary complications, like pneumonia and bronchiolitis, often occur after acute viral infections, and thus viral shedding may be missed by the time of hospitalization.	In addition to molecular diagnostics, serologic assays will be used to identify recent influenza and RSV infections.
More information is needed on the virus-specific attributable fraction for influenza and RSV disease.	Assess the prevalence of influenza and RSV infections among healthy infants who have not been ill for at least 7 days at specimen collection (all study years) and confirm absence of symptoms up to 4–10 days after collection (starting in year 2 and continuing afterwards).
B. Describe the clinical features of influenza- and RSV-associated hospitalizations among infants and the predictors of very severe disease	
Knowledge Gap	Study Feature
Information is limited on the non-respiratory disease manifestations of influenza and RSV infections among infants.	Assess the frequency of influenza and RSV infections among infants hospitalized with non-respiratory illness (including febrile seizures, otitis media, diarrhea, and sepsis-like syndromes).
The range of clinical severity for influenza and RSV infections among infants is poorly characterized outside of high-income countries.	Examine symptoms and signs (including temperature, oxygen saturation, and respiration), oxygen support, and treatments at admission and then daily during hospitalization for influenza and RSV infected infants.
Extent to which antibiotics may be over-utilized and influenza antivirals may be under-utilized among infants is unclear, especially outside of high-income countries.	Describe the use of and timing of administration of antibacterial and antiviral agents during infants’ hospitalization.
Further research is needed to identify risk factors for very severe disease (i.e., requiring intensive care), especially outside of high-income countries.	Assess the characteristics of infants (e.g., age, sex, prematurity, co-morbid conditions), viruses, and environmental characteristics (e.g., socio-economic status, household composition, distance from hospital) associated with more severe illness presentation.
Information on the clinical course of influenza and RSV infections during and following hospitalization is limited, especially outside of high-income countries.	Describe the length of stay in the general ward or ICU and the frequency of death and hospital re-admission within 30 days post-discharge among enrolled infants.
C. Describe the acute antibodies to influenza and RSV by months of age among infants <1 year old and their humoral immune response to infections.	
Knowledge Gap	Study Feature
Given that infancy is a period of dynamic immune system development, there is limited information on the antibody response of infants.	Describe the influenza and RSV antibody response of infants by age sub-strata for all infants at hospital admission.
More information is needed on the prevalence of influenza antibodies among infants of mothers who received influenza vaccination during pregnancy.	In study sites where influenza vaccination is available, describe the influenza antibody profiles of infants born to influenza vaccinated vs. unvaccinated mothers.
Implications of pre-infection influenza and RSV antibodies to the manifestation of disease and immune response among infants are unclear (especially those aged <6 months).	Compare acute antibodies for influenza and RSV (at hospital admission) and subsequent sero-conversion among infants receiving general vs. intensive care by age sub-strata.
Information on the frequency with which influenza and RSV infections result in robust antibody response is limited, especially by sub-age-strata among infants aged <1 year.	Describe the frequency of serologic conversion to influenza and RSV using acute and convalescent sera among infants with infections confirmed by real-time reverse transcriptase polymerase chain reaction (rRT-PCR) assay.

## Methods/Design

This prospective study consists of a hospital-based cohort and a cross-sectional sample of non-ill infants enrolled over 3 years in hospitals and ambulatory care settings serving infants in four countries (Table 2).^3^ All study sites use a common protocol and data collection tools (using personal computers or mobile devices).^4^ The design and screening methodologies for the hospital and community studies are described separately (below) followed by a description of common laboratory, statistical, and data management procedures.

**Table 2:** Network study countries, sponsors, and enrollment sites

City, Country (Local Population Served by Hospitals)	Sponsoring Institution	Hospitalized Infant Study			Non-Ill Infant Study
		Study Hospitals	Number of Pediatric General Ward Beds	Number of Pediatric Intensive Care Beds	Non-Ill Enrollment Sites
Tirana, Albania (~610,000)	South East European Center for Surveillance & Control of Infectious Diseases	Pediatric Department University Hospital “Mother Theresa”	88	25	Enrolled during well-baby immunization visits to (a) Mother and Child Consultancy Room, Health Center No. 4 Tirana, Tirana Regional Health Authority, and (b) the Pediatric Surgical Ward, University Hospital “Mother Theresa,” Tirana, Albania
		Maternity Hospital “Queen Geraldine” Neonatology Unit	19	5	
Amman, Jordan (~4 Million)	The Eastern Mediterranean Public Health Network	Al-Basheer Hospital, Maternal and Pediatric Building	120	70	Enrolled from Al-Owdah Primary Healthcare Center, which provides maternal and child health services; infants are recruited during routine visits for immunization, growth monitoring, or other well-baby check-ups
Managua, Nicaragua (~1 Million)	Sustainable Sciences Institute	Hospital Infantil Manual De Jesus Rivera “La Mascota”	270	31	Enrolled from Health Center Socrates Flores Vivas during immunization visits, well-baby check-ups, and from a local pediatric cohort study, previously described [34, 35]
Bohol Island, The Philippines (~1.4 Million)	Research Institute for Tropical Medicine	Governor Celestino Gallares Memorial Regional Hospital	42	8	Enrolled during immunization visits at (a) Cogon Lower Barangay Health Station, (b) Cogon Upper Barangay Health Station, and (c) Taloto Health Center

### Hospitalized infants study design

**Fig. 1:**
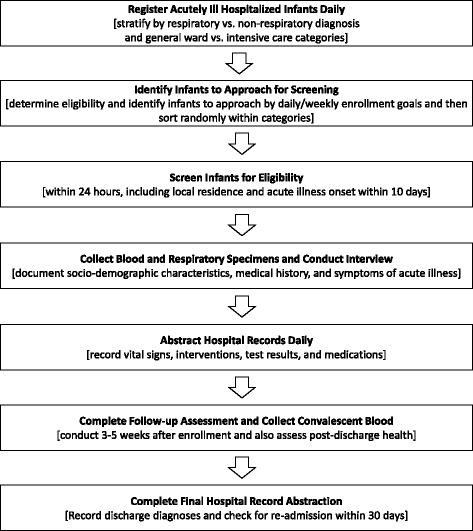
Steps in Hospital Enrollment and Follow-up

For each study year, each site is enrolling approximately 350–500 infants aged <1 year old hospitalized with an acute medical illness during periods of local influenza circulation in year 1 and during periods of either influenza viruses or RSV circulation starting in year 2 (and for any subsequent years of the study), as indicated by local and national virus surveillance platforms.^5^ We intend to begin enrollment during the early phase of local influenza virus and RSV circulation and continue past the peak through the decline in local circulation if possible. Enrollment goals are set per week in order to sample infants and extend the use of resources across the study period; enrollment can potentially expand during periods of peak circulation of either influenza or RSV.^6^ Each day, study staff use hospital admission records (including presenting complaints and preliminary admission diagnoses) to register all acutely ill infants and stratify them into respiratory versus non-respiratory illness groups and general ward versus intensive care unit (ICU) placements (Fig. 1).^7^ Infants within the resulting four strata are randomly sorted and prioritized in order to approach a mixture of patient types until the maximum daily enrollment in each group has been met. 8 Infants are screened for eligibility within 24 h of hospital admission to confirm that the family lives in the local area and that the illness onset was within the prior 10 days. Only basic descriptive information is then recorded for infants who are ineligible, are not approached, or whose parents refuse informed consent; these data will be used to assess possible selection bias and to describe the denominator of weekly hospitalized acutely ill infants. Study staff collect other hospital census information daily, including the number of available pediatric general ward and ICU beds, to consider differences in access to hospital care over time and between study sites.

After completing written informed consent, extensive socio-demographic [28, 29], epidemiological, and clinical information is collected at enrollment, at a 3–5 week follow-up encounter, and through hospital record abstraction (summarized in Table 3). Respiratory specimens using combined nasal and oropharyngeal (OP) swabs (or endotracheal aspirate for intubated infants) and up to 2.5 ml of blood are collected by trained staff within 24 h of admission.^9^ Information on socio-demographic characteristics, medical history, maternal and infant vaccination history (documented from vaccination cards when available), and symptoms of the acute illness are gathered from parents (or guardians).

**Table 3:** Key Variables and Sources of Information for Hospitalized and Non-Ill Infants

	Hospitalized Infants			Non-Ill Infants
	Enrollment	3–5 Week Follow-Up	Hospital Records	Enrollment
Specimens				
Combined nasal and oropharyngeal swabs	√			√
Blood (Sera)	√	√		
Endotracheal aspirate (for intubated infants)	√			
Infant illness				
Date of illness onset and resolution	√	√		
Maternal-reported symptoms	√	√		
Ambulatory and self-care for illness	√	√		
Clinical signs at admission and per day			√	
Clinical diagnoses			√	
Clinical interventions			√	
Clinical laboratory results			√	
Clinical radiographic information			√	
Readmission to hospital		√	√	
Other hospitalizations post-discharge		√	√	
Infant characteristics				
Demographic (sex, birth date, race, ethnicity) information	√			√
Current weight	√	√	√	√
Current length	√	√	√	√
Perceived overall health and functioning	√	√		√
Influenza vaccination status (from vaccination cards when available)	√	√	√	√
Delivery characteristics (including gestational age, birth weight, complications and abnormalities)	√		√	√
Diagnoses and history of care for chronic conditions	√		√	√
Diagnoses and history of care for prior acute illnesses	√		√	√
Results of previous laboratory tests			√	
Other immunization status	√	√	√	√
Daycare attendance	√			√
Household characteristics				
Age of each household member	√			√
Education and occupation of parents (or guardians)	√			√
House size and number of rooms	√			√
Subjective social status	√			√
Household wealth index	√			√
Household smoking	√			√
Maternal characteristics				
Subjective health status	√	√		√
Medical conditions	√			√
Pregnancy history and complications during study infant’s gestation	√			√
History of and current breastfeeding	√			√
Influenza vaccination status (from vaccination cards when available)	√	√	√	√
Knowledge of and worry about influenza illness		√		√
Knowledge of and attitudes toward influenza vaccine for infants aged ≥6 months		√		√

Hospital records are abstracted to document clinical signs and measurements at admission, including clinician-measured temperature, respiration rate, and oxygen saturation. For each subsequent day of hospitalization, we document clinical signs and the lowest and highest measurements of vital signs and oxygen saturation. Clinical diagnoses at admission and discharge are recorded, including International Classification of Diseases (ICD) codes for discharge diagnoses. Clinical interventions, such as oxygen support, and treatments (including antibacterial and antiviral agents) are documented throughout hospitalization. Clinical laboratory values (such as white blood cell count) and test results (including bacterial, viral, and parasite detection), chest radiographic information (including digital images, if available), and other clinical assessments carried out as part of regular clinical care are also recorded. We also document hospital readmissions within 30 days of discharge for the index illness.

All infants in the hospital-based cohort complete a follow-up assessment and a convalescent blood draw (up to 2.5 ml of blood) 3–5 weeks after enrollment through hospital or home visits. Information on the infant’s health since hospital discharge (including recovery, readmission, or death) are gathered from parents and may be supplemented by review of medical and administrative records. At the follow-up assessment, questions about knowledge, attitudes, and practices (KAP) toward influenza illness, including the extent of worry about influenza [30], are asked for all parents; KAP toward influenza vaccines are asked among parents of infants aged >6 months, including previously validated measures of intention to vaccinate [31–33] and the perceived effectiveness and safety of influenza vaccines [31, 33].

### Community non-ill infants study design

For each study year, concurrent with hospital enrollment, approximately 150–300 non-ill infants aged <1 year per site are enrolled during routine preventive care encounters with weekly enrollment goals stratified by ages 0–5 months and 6–11 months in order to reflect the mixture of ages observed among hospitalized infants. As described in Table 2, all study sites enrolled non-ill infants during routine immunization visits; Jordan also enrolled infants during routine growth monitoring and other well-baby check-up visits; Nicaragua enrolled additional non-ill infants from an existing prospective cohort study [34, 35]. Screening questions confirm that the infant has not been acutely ill within the past 7 days with nasal congestion or discharge, new or worsening cough, shortness of breath, difficulty breathing, wheezing, fever, chills, or diarrhea. After informed consent is obtained from parents, trained staff collect combined nasal and OP swabs and complete a structured interview covering the same infant, household, maternal characteristics, and KAP assessed for hospitalized infants (Table 3). No blood is collected from non-ill infants. Starting in the second year of the study, a follow-up contact will be made to parents of non-ill infants (4–10 days after enrollment) to assess whether the infant developed illness symptoms after the initial interview.

### Laboratory methods

Respiratory specimens are tested for influenza and RSV by reference laboratories at each study site using validated singleplex real time reverse transcriptase polymerase chain reaction (rRT-PCR) assays, with protocols, primers, probes, and reagents supplied by US CDC.^10^ Study laboratories completed WHO influenza proficiency panels and quality assurance testing (of every 5^th^ influenza positive and every 20^th^ influenza negative) administered by Marshfield Clinic Laboratory (Marshfield, Wisconsin). RSV proficiency panels were administered and reviewed by US CDC.

Also at each study site’s research laboratory, we will complete multiple virus testing on respiratory specimens from both hospitalized and non-ill infants using multiplex real-time PCR for detection of pathogen genes by TaqMan® technology (Fast-track Diagnostics [FTD], Silema, Malta); specifically, we are utilizing FTD Respiratory Pathogens 21, which is a five tube multiplex for detection of influenza A virus, influenza A(H1N1) swine virus, influenza B virus, rhinoviruses, coronaviruses (NL63, 229E, OC43, HKU1), parainfluenza viruses 1-4, human metapneumovirus, bocavirus, respiratory syncytial virus, adenoviruses, enteroviruses, parechoviruses, *Mycoplasma pneumoniae*, and an internal control. Prior to beginning multiplex testing, each site laboratory completed Respiratory II EQA (External Quality Assessment) Programme by QCMD (Glasgow, Scotland; RESPII16 Catalogue Number QAV164189), which includes molecular detection and determination of human metapneumovirus, respiratory adenoviruses, rhinoviruses, coronaviruses and parainfluenza viruses. Although we will compare influenza and RSV results from both singleplex and multiplex assays, the singleplex rRT-PCR results will be the “gold standard” or primary assay.

A random subset of all influenza viruses may undergo virus isolation, depending on the availability of virus characterization information from local surveillance platforms. Also, some influenza viruses/specimens may have molecular characterization with genetic sequencing and other assays to detect genetic markers and antiviral resistance. A sampling of specimens positive for RSV will be selected and characterized by target genetic sequencing. Remaining aliquots of all study respiratory specimens will be sent to a CDC-designated facility for banking and storage; no specimens will contain personal identifiers. These specimens may also be utilized to investigate biomarkers of disease severity or novel viruses or pathogens.

Hemagglutination inhibition (HI) assay will be completed following the US CDC protocol [36] at the Battelle laboratory (Aberdeen, Maryland), after completing a proficiency panel. Duplicate results will be used to calculate the geometric mean antibody titer (GMT) against seven influenza A and B virus strains that include local influenza vaccine strains and circulating strains for the study year (for possible infant exposure) and prior years (for possible maternal exposure). Serologic confirmation of influenza infection will be defined as ≥4 fold rise in GMT from acute to convalescent sera, with convalescent HI titer of ≥ 40 GMT.

RSV-specific serum IgG, IgM, and IgA will be detected using standard enzyme-linked immunosorbent assay (ELISA) procedures [37, 38]. Endpoint titers will be determined for serum antibodies to the F and G proteins of RSV.

### Data management

Data collection and site-level management are conducted using REDCap (Research Electronic Data Capture), which is a browser-based metadata-driven software system (Vanderbilt University, Nashville, TN) [39]. The recruitment log (including randomization and prioritization by recruitment strata), screening survey, informed consent, enrollment interview, specimen management, laboratory tracking and results, daily hospital abstraction, and follow-up assessment occur via REDCap forms on personal computers or mobile devices (tablets or smart phones).

Routine quality assurance monitoring is conducted at each study site and centrally by the data coordinator (Abt Associates). Missing or unclear information is corrected through follow-up contact with parents or medical record abstraction. Chained imputation methods or other methods will be used for imputing missing covariate data (i.e., not primary outcomes), if necessary.

### Statistical considerations

Sample needs for the objectives to assess the percentage of influenza and/or RSV positives (by molecular or serologic methods) are driven by the precision with which we wish to measure the lowest expected percentage of infection for a particular category of infant hospitalizations at a specific study site. For optimum precision, we intend that the 95% confidence interval (CI) is within the range of plus or minus 50% of the observed percentage positive. For example, in order to measure a percentage of rRT-PCR confirmed influenza of 5% (with a 95% CI from 2.5–7.5%) within a particular patient strata would require a sample size of 328 (and 16 positives) [40]. Thus, we expect to make site-specific estimates for large infant categories (e.g., respiratory vs. non-respiratory illnesses). However, it will be necessary to combine results across study sites in order to estimate positivity for combinations of categories (e.g., non-respiratory illnesses among infants aged 0–5 months in ICU).

Sample goals for the non-ill infants were originally made with the expectation of observing at least 2% of infants positive for influenza virus and RSV infections [27, 41–43]. We recognized that 200 non-ill infants would be needed to observe a 2% rate of virus positivity with minimum precision (with a 95% CI = +/- 1.9%). Estimation of the attributable fraction of disease associated with influenza or RSV will require statistical adjustment and/or matching of ill and non-ill infants by season, weeks within season, age, and characteristics that may differ between infants recruited from preventive care settings and the source population [27]. The proposed 150–300 non-ill enrollees per year may not be adequate to capture this variation adequately.

### Ethical approval & ethical considerations

The study protocol and procedures have been reviewed and approved by Institutional Review Boards (IRBs) at each study site and by Abt Associates (the coordinating institution on which US CDC relies). Written informed consent is completed in the parent or guardian’s language (Spanish, Arabic, Albanian, or Visayan [Boholano dialect]).^11^ Provision of a small gift or travel reimbursement vary by study site depending on local IRB guidelines. Whenever possible, blood collection is coordinated with clinically necessary blood draws. Given the research nature of the laboratory methods and time delays in batch testing, rRT-PCR findings are not available to inform clinical decisions.

## Discussion

We described the recruitment, data gathering, laboratory, and follow-up procedures for this prospective study of approximately 3,000 hospitalized infants and 1,400 non-ill infants aged <1 year as part of the US CDC-funded IRIS network. Estimating the hospital burden of severe infant disease associated with influenza and RSV and expanding our understanding of the clinical presentation and antibody response associated with these diseases are especially timely and relevant aims given existing and new immunoprophylaxis options for RSV [44, 45], the existing availability of influenza vaccines, and the development of new types of influenza and RSV vaccines and vaccine strategies to provide direct or indirect protection for infants [46–49].

### Study strengths

By focusing on all acute infant illnesses, our prospective study promises to quantify the proportion of severe influenza and RSV disease that is missed by traditional surveillance approaches, case definitions, and molecular diagnostics. It is unique in its systematic sampling of acutely ill infants that does not rely on clinical judgment, its collection of in-depth clinical and socio-demographic information, and its use of both molecular and serologic diagnostics. Our Network extends research beyond the USA and other high-income countries and also includes countries with prolonged or multiple epidemic periods (Nicaragua and the Philippines), which are also under-represented in influenza and RSV research. Other strengths include the multi-country composition of our network, which will allow us to examine the consistency of trends across settings with different medical systems and infant populations. In addition, given the multi-agent source of many infant diseases [27, 50–55] and the absence of community controls in many hospital studies [27], the enrollment of non-ill infants from the same community is an important study feature which will allow us to examine the frequency of influenza virus and RSV infections among non-ill infants and, depending on sample size and virus attack rate, possibly estimate the virus-specific attributable fraction of influenza and RSV to severe disease.

### Limitations

Our study has at least six limitations. First, the number and types of influenza virus infections we identify will depend on the circulating viruses in each country during the years of our study and may under- or over-estimate the expected frequency of disease in future years. Indeed, reviews of influenza studies in Africa and India observed 5- to 20-fold differences in incidence estimates across studies [56, 57]. Similarly, due to the timing and funding cycle of the study, we may not enroll infants during complete influenza and RSV seasons in all countries for all study years.

Second, different hospitals and medical systems likely have different clinical thresholds for hospitalization [6, 27, 58, 59], which will make comparisons across settings difficult; yet, this may be less of a concern when we examine the subset of infants requiring intensive care, who would likely be admitted in most settings. Third, the IRIS network does not include a low income country. Therefore, our ability to generalize from the middle-income countries in the IRIS network to low-income countries, where the burden of infant disease is high and access to hospital care is poor, is limited.

Fourth, although we expect our study methods will increase the identification of influenza and RSV infections, we recognize that our observations will continue to underestimate the true burden of these diseases; for example, serologic diagnostic assays will fail to identify some infections due to known limits in the measurement sensitivity of HI [60–62] and the inability of immune challenged infants to mount a robust antibody response [63–65]. Fifth, as an observational study that relies on existing local clinical practices, we will not have standardized measures of vital signs or clinical laboratory values using common procedures and calibrated instruments, which will limit the validity, reliability, and comparability of these indicators. Sixth, our focus on viral etiologies excludes description of other infectious and non-infectious etiologies of acute illness.

## Conclusions

The IRIS network promises to expand our knowledge of the frequency, clinical features, and antibody responses associated with serious influenza and RSV disease among infants aged <1 year. Although variations in findings across study sites and years is a common challenge [66], we hope to identify at least two consistent trends regarding influenza among infants: (i) the proportion of non-respiratory hospital admissions among infants that are associated with influenza infections; (ii) the proportion of influenza infections that are missed by molecular diagnostics but can be identified by serologic diagnostics. For RSV, we aim to: (iii) identify risk factors associated with severe RSV disease across countries; (iv) evaluate the RSV-specific antibody response among infants hospitalized with severe RSV infections. These insights could aid countries in making more precise estimates of disease burden and inform decisions about the potential value of existing and new vaccines and other prevention and treatment strategies.

## Abbreviations

GMT: Geometric mean titer
HI: serum hemagglutination inhibition antibody (assay)
ICU: intensive care unit
IRB: Institutional review board
IRIS: influenza and RSV in infants study
rRT-PCR: real time reverse transcriptase polymerase chain reaction assay
RSV: respiratory syncytial virus
US CDC: Centers for Disease Control and Prevention (USA)

## Footnotes

1 The duration of viral shedding among infants may be less of a concern for RSV infections, where prolonged viral shedding has been observed in studies of symptomatic children (Munywoki, et al. [67]).

2 Study planning began at a workshop on April 15–16, 2015 in Atlanta, Georgia USA attended by site principal and co-investigators and investigators and advisors from US CDC and Abt Associates. A second planning workshop by investigators to review results of the first year of enrollment and make minor revisions to protocols and procedures took place in Tirana, Albania on April 25–28, 2016.

3 All sites have completed year 1 enrollment within the following enrollment dates: Nicaragua (6/26/2015–1/29/2016), Philippines (10/1/2015–9/30/2016), Albania (12/1/2015–5/2/2016), and Jordan (12/27/2015–5/2/2016).

4 The Philippines site changed their primary enrollment sites during several months in 2016 in the following ways. During June to September, 2016, the Philippines site attempted to expand enrollment of hospitalized infants at four small hospitals on Bohol Island (Borja Family Hospital, Ramiro Community Hospital, Tagbilaran Community Hospital, and Tagbilaran Maternity & Children’s Hospital). Given the relatively small number of eligible infants hospitalized in these settings, the site returned to enrolling infants only from the primary hospital (Governor Celestino Gallares Memorial Regional Hospital) starting in October, 2016. Also, during the initial months of the study (October to December, 2015), the site attempted to enroll non-acutely ill infants scheduled for routine surgical procedures at the primary study hospital, but abandoned this approach due to the relatively small number of eligible non-ill infants that could be enrolled from this setting.

5 Specifically, Albania plans to enroll during November through May for each study season based on historical patterns of seasonal influenza circulation as documented by national sentinel surveillance systems for Severe Acute Respiratory Illness (SARI) and Influenza-like Illness (ILI) at the Institute of Public Health. Jordan plans to enroll during December through April for each study season, with the start of enrollment also informed by reports of rRT-PCR confirmed influenza cases from the Central Public Health Laboratory of Jordan through their sentinel surveillance networks. Nicaragua enrolled from June through February in the first year, based on historical influenza data from two ongoing pediatric cohort studies in Managua, previously described (Gordon, 2015; Gresh, 2016); starting in year 2, enrollment began a month earlier, based on historical surveillance data that indicated RSV circulation began as early as May. The Philippines plans to continue enrollment in year 1 through December, 2016, based on indicators of multiple influenza epidemic periods as documented by WHO FluNet surveillance; additional data on influenza and RSV circulation was referenced from the National Influenza Center at the Research Institute for Tropical Medicine in Manilla.

6 For example, Nicaragua implement a three-tiered enrollment plan in year 1 whereby enrollment increased when either (1) >5% of cohort samples are influenza positive or (2) 5% of national surveillance samples from Managua are influenza positive. Enrollment targets ranged from 4–24 hospitalized infants per week and 0 to 12 non-ill infants per week. Enrollment decreased one week following influenza positivity rates dropping below 5% among samples from the community cohort, national surveillance platforms, and study hospital. In year 2, enrollment will increase when either (1) >5% of cohort samples are influenza positive, (2) >5% of national surveillance samples are influenza positive, (3) >5% of study samples are influenza positive, or (4) >10% of study samples are RSV positive. Enrollment targets will range from 6–28 hospitalized infants per week and 3–11 non-ill infants per week. Enrollment will decrease when none of the preceding conditions are met.

7 Exclusion criteria including (a) Residence is outside of local area and thus unable to participate in follow-up; (b) Parent or guardian is not available to provide information on the infant’s index illness or medical history; (c) Parent or guardian is unable to understand informed consent due to language or other communication barrier; (d) Infant is presenting for a non-acute illness such as injuries, congenital ailments, or chronic conditions (without an acute onset date); (e) Infant has not been discharged from the hospital after delivery.

8 Specific clinical admission diagnoses monitored by all sites include respiratory illnesses (e.g., acute respiratory distress syndrome, asthma exacerbation, bronchiolitis, pertussis-like syndrome, pneumonia, reactive airway disease exacerbation, respiratory failure) and non-respiratory illnesses (e.g., bacteremia, dehydration, encephalitis, febrile seizures, meningitis, sepsis, urinary tract infection). At admission, these are preliminary clinical diagnoses or symptom and syndromic descriptions; they are not captured as ICD codes at study sites during the period when enrollment occurs. Acute illnesses can be categorized as involving respiratory only, non-respiratory only, or both respiratory and non-respiratory presentations. For the purposes of stratification and sampling, infants with both respiratory and non-respiratory illness indications are counted in the respiratory category. In this way, we prioritize enrollment of infants with non-respiratory presentations given the importance of enrolling infants with atypical influenza or RSV disease. This initial categorization only serves to prioritize screening; complete signs, symptoms, and diagnoses are recorded for all enrolled infants.

9 Nasal and oropharyngeal swabs are ULT Minitip flock swabs (Copan Diagnostics, Inc., Murrieta, CA USA). After collection, they will be combined in the same tube with MicroTest Room Temperature transport medium from Remel Products (Lenexa, KS USA).

10 Site laboratories are Institute of Public Health Tirana (Albania), Central Public Health Laboratory Jordan, Centro Nacional de Diagnostico y Referencia (CNDR) (Nicaragua), and Research Institute for Tropic Medicine (Philippines). All laboratories are WHO National Influenza Centers.

11 English translations of the informed consent documents from each study site, approved by Institutional Review Boards at study sites and at Abt Associates, are available from the authors.
